# Application of myocardial work in predicting adverse events among patients with resistant hypertension

**DOI:** 10.1186/s13019-023-02468-y

**Published:** 2023-12-06

**Authors:** Limin Luo, Yongshi Wang, Huiping Hou, Qiang Liu, Zehan Xie, Qiaoyan Wu, Xianhong Shu

**Affiliations:** 1 Department of Echocardiography, Zhongshan Hospital (Xiamen), Fudan University, Xiamen, Fujian China; 2 Department of Echocardiography, Xiamen Municipal Clinical Research Center for Medical Imaging, Xiamen, Fujian China; 3 Department of Echocardiography, Xiamen Clinical Research Center for Cancer Therapy, Xiamen, Fujian China; 4 Department of Echocardiography, Shanghai Institute of Medical Imaging, Shanghai Institute of Cardiovascular Disease, Zhongshan Hospital, Fudan University, Shanghai, China

**Keywords:** Myocardial work, Strain, Echocardiography, Hypertension, Resistant hypertension

## Abstract

**Background:**

Hypertension is the most common chronic disease and the leading risk factor for disability and premature deaths worldwide. Approximately 10–20% of all patients with hypertension and 15–18% of the general population who are treated for hypertension have resistant hypertension (RH). Patients with RH have a higher risk of end-organ damage, such as carotid intima–media thickening, retinopathy, left ventricular hypertrophy and heart failure, myocardial infarction, stroke, impaired renal function, and death than those with controlled blood pressure. In the present study, we applied echocardiography to patients with RH to evaluate myocardial work (MW) and determine whether it is predictive for the occurrence of adverse events within 3 years.

**Methods:**

We included 283 outpatients and inpatients aged ≥ 18 years who met the clinical criteria for RH, without arrhythmia and severe aortic valve stenosis, between July 2018 and June 2019. The patients were followed up for 3 years from starting enrollment, and any adverse event that occurred during the period was used as the observation end point. Each enrolled patient underwent a complete transthoracic echocardiogram examination, blood pressure was measured and recorded, and MW was then analyzed.

**Results:**

Eighty-two (28.98%) patients with RH had adverse events, such as myocardial infarction (n = 29, 35.36%), heart failure (n = 4, 0.05%), renal insufficiency (n = 40, 48.78%), renal failure (n = 2, 0.02%), cerebral infarction (n = 5, 0.06%), and cerebral hemorrhage (n = 2, 0.02%), and no death events occurred. In patients with RH and adverse events, global longitudinal strain (GLS) (− 16% vs. − 18%), the global work index (2079 mmHg% vs. 2327 mmHg%), global constructive work (2321 mmHg% vs. 2610 mmHg%), and global work efficiency (93% vs. 94%) were lower than those in patients without adverse events. However, global wasted work (GWW) was higher in patients with RH and adverse events than in those without adverse events (161 mmHg% vs. 127 mmHg%). GLS and GWW were the most significant in predicting adverse events.

**Conclusions:**

MW, especially GLS and GWW, is a good method to predict 3-year adverse events in patients with RH.

**Graphical Abstract:**

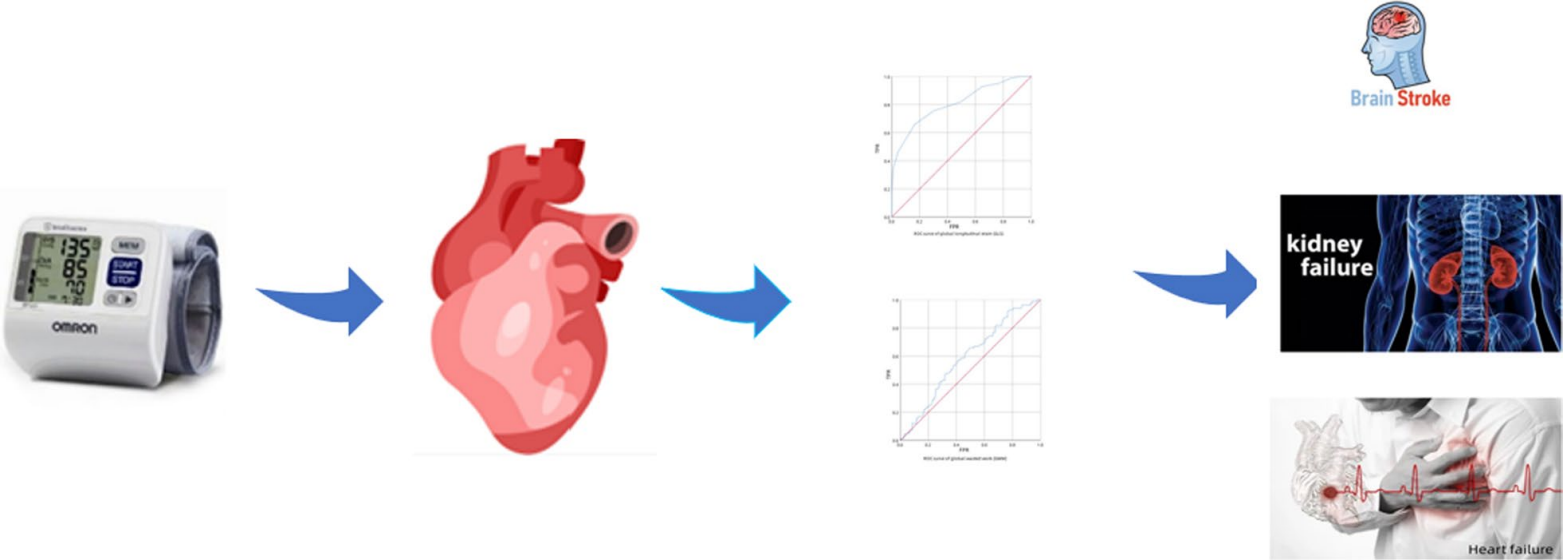

## Background

Hypertension affects approximately one billion adults. Hypertension accounts for approximately 9% of global disability-adjusted life years and is associated with more than nine million deaths annually [1, 2]. On the basis of observational studies, each 10 mmHg increase in systolic blood pressure (SBP) is associated with a 45% higher risk of ischemic heart disease and a 65% higher risk of ischemic or hemorrhagic stroke in those aged 55–64 years [3, 4].

Resistant hypertension (RH) is defined as the failure to achieve recommended clinic (office) blood pressure (BP) goals, despite the concurrent use of three antihypertensive medications of different classes at optimal dosages These medications commonly include a long-acting calcium channel blocker, a blocker of the renin–angiotensin system (an angiotensin-converting enzyme inhibitor or angiotensin receptor blocker), and a diuretic, or achieving BP goals with four or more drugs after causes of pseudoresistance are ruled out [5]. The prevalence of RH ranges from 10 to almost 30% in hypertensive patients [6]. Patients with RH have a higher risk of end-organ damage, such as carotid intima–media thickening, retinopathy, left ventricular hypertrophy and heart failure, myocardial infarction, stroke, impaired renal function, and death than those with controlled BP [7–9]. In a retrospective study of > 200,000 patients with incident hypertension, those with RH were 47% more likely to suffer the combined outcomes of death, myocardial infarction, heart failure, stroke, or chronic kidney disease over a median of 3.8 years of follow-up [8]. In another study of > 400,000 patients, patients with RH had a 32% increased risk of developing end-stage renal disease, 24% had an increased risk of an ischemic heart event, 46% had an increased risk of heart failure, 14% had an increased risk of stroke, and 6% had an increased risk of death compared with patients without RH [10]. RH includes the following three categories [11]. (1) Pseudo-RH is not true RH. Pseudo-RH occurs because of inaccurate measurement of BP, medication nonadherence, or the white coat effect. (2) In true RH, pseudo-RH is excluded and it meets the 2018 American Heart Association diagnostic criteria for RH. (3) Apparent treatment RH is defined as when one or more of the following elements are missing: medication dose, adherence, or out-of-office BP. Therefore, pseudoresistance cannot be excluded [12].

Adverse events associated with hypertension comprise all fatal and nonfatal cardiovascular events. These events include fatal and nonfatal acute myocardial infarction, sudden cardiac death, new-onset heart failure, death from progressive heart failure, any myocardial revascularization procedure, fatal and nonfatal stroke, any aortic or lower limb revascularization procedure, any amputation above the ankle, death from aortic or peripheral arterial disease, the beginning of dialysis, and death due to renal failure [13].

This study aimed to investigate a method and related indicators that can be used to predict the occurrence of adverse events in patients with RH. Increased cardiac afterload affects the myocardium owing to chronic hypertension. Therefore, we decided to apply myocardial work (MW) and related indicators evaluating by echocardiography in patients with RH to determine which indicators could be used to predict the occurrence of adverse events. This method was first proposed by Russell and his colleagues, and is a non-invasive echocardiographic method based upon an estimated left ventricular (LV) pressure curve in combination with strain by speckle-tracking echocardiography (STE) [14]. They found that the LV pressure–strain loop area using the non-invasive LV pressure curve showed a strong correlation and a good agreement with the loop area using invasive LV pressure when they applied this method to patients with left bundle branch block. They also compared ischemic versus non-ischemic segments’ non-uniformity in work distribution and showed that the non-invasive pressure–strain loop area reflected regional metabolism [14]. This method takes into account deformation and afterload.

## Material and methods

### Patient population and study design

This was a single-center, prospective, observational study that included 283 outpatients and inpatients aged ≥ 18 years who met the clinical criteria for RH without arrhythmia and severe aortic valve stenosis. These patients had regular therapy at Zhongshan Hospital (Xiamen), Fudan University between July 2018 and June 2019 and were followed up for 3 years. At the same time, we selected the same number of non-hypertensive outpatients or inpatients with a similar sex and age composition as controls. Patients with RH who had adverse events during the follow-up were included in the independent group, while patients with RH who did not have adverse events were included as controls. The indices related to global MW in patients with RH at the time of enrollment were retrospectively analyzed. The differences in correlation parameters of MW between the two groups were compared to obtain some useful indices for predicting adverse events in patients with RH. Exclusion criteria were as follows: patients < 18 years old, those with pseudo-RH, arrhythmia, severe aortic valve stenosis, or poor image quality, patients who have been diagnosed with obstructive sleep apnea (OSA), those who refused to participate in the study, and those who could not sign a written consent form.

### Transthoracic echocardiography

Comprehensive transthoracic echocardiography (TTE) was performed by an experienced sonographer using a Vivid E95 ultrasound system equipped with an M5S 3.5-MHz transducer (GE Vingmed Ultrasound, Horten, Norway) with analysis software (EchoPAC version 203; GE Vingmed Ultrasound). All patients underwent a complete TTE examination. Patients were scanned in the left lateral decubitus position and connected to an electrocardiogram. All two-dimensional (2D) and Doppler recordings and measurements were performed according to American Society of Echocardiography guidelines [15]. Two-dimensional images included the LV long-axis view, LV short-axis view, short-axis view of the artery, and the apical two-, three-, and four-chamber views (frame rate of 50–90 s^−1^). We used the LV short axis view to measure LV wall thickness, and used the apical two-, three-, and four- chamber views to analyze LV global MW according to the suggestions by Russel et al. [14].

### Myocardial work

Myocardial work (MW) classically has been calculated as the area of pressure–volume loop of the left ventricle (LV), following the brilliant pioneering ideas of Otto Frank at the end of the nineteenth century and later of Hiruyuki Suga and Kiichi Sugawara, who modeled work done by the LV as an extension of Hooke’s law of the elasticity of a spring and introduced the concept of time-varying elastance [16]. An experimental study showed the area of the LV pressure–volume loop reflects stroke work as well as myocardial oxygen consumption, and it was later confirmed that this concept is valid clinically [16, 17]. According to the same principle, the area of the myocardial force–segment length loop reflects regional myocardial work and oxygen consumption [18]. MW assessment was initially calculated using invasive pressure measurements, which limited its widespread use in clinical practice [19, 20]. In 2011, Russell [14] and his colleagues first proposed a new non-invasive echocardiographic method based upon an estimated LV pressure curve in combination with strain by speckle-tracking echocardiography (STE). They found that LV pressure–strain loop area using the non-invasive LV pressure curve showed a strong correlation and a good agreement with loop area using invasive LV pressure when they applied the method to the patients with LBBB and compared ischaemic vs. non-ischaemic segments’ non-uniformity in work distribution was also apparent, at the same time, showed non-invasive pressure–strain loop area reflects regional metabolism [14]. This method takes into account deformation as well as afterload.

MW is composed of the following parameters: (1) Global MW index (GWI): total work within the area of the LV pressure–strain loop and is calculated from mitral valve closure to mitral valve opening. (2) Constructive MW: work performed by the LV contributing to LV ejection during systole. Constructive MW is defined as shortening of myocytes during systole and lengthening of myocytes during isovolumic relaxation. (3) Wasted MW: work performed by the LV that does not contribute to LV ejection. Wasted MW is defined as myocardial work during lengthening in systole {adding shortening during isovolumic relaxation (IVR)}. (4) MW efficiency: constructive MW/(constructive MW+wasted MW) [21].

### MW analysis

The analysis of MW was performed mainly through the analysis software of the instrument or was performed offline with the same software. GLS analysis was the main step in the analysis of MW because GLS represents the displacement of LV myocardial deformation. We used 2D speckle-tracking to acquire the GLS. In this process, event timing was the first important step. Most speckle-tracking software packages use an electrocardiogram (R-wave trigger for defining the zero reference of the strain curve). However, this method is only applicable when QRS is normal, and if conduction delay occurs, the zero reference of the strain curve will be incorrectly defined [22]. The best option for this situation is for the definition of end-diastole and end-systole to be adjusted manually. The second important step is the definition of the region of interest (ROI), we should make the center line moves with the source 2D image. After obtaining these relevant data, the instrument provided the corresponding pressure–strain curve and a bull’s eye diagram. Therefore, we could visualize how much work the heart was doing overall. We also obtained the MW-related data of each segment. In addition to the data of the global myocardial work, the MW correlation parameters of the segments had refined some indicators of the global work and added some indicators.

### Follow-up

All subjects were followed up with telephone conduction each 3 moth and interview conduction each 6 moths. The observation period for each subject was the number of months from the baseline evaluation to the date of the last clinical visit or the first end point, whichever came first. Any fatal or nonfatal adverse events happened was regarded as the end point. End points were adjudicated from medical records, and interviews with the attending physicians and patients’ families, using a standard questionnaire reviewed by an independent observer.

### Statistical analysis

Statistical analysis was performed using IBM SPSS software (version 26.0; IBM Corp.). Continuous data are expressed as the mean and standard deviation or median with the 25th–75th interquartile range (IQR), and categorical data are expressed as the percentage and frequency. Differences between the two types of continuous data consistent with a normal distribution were compared by the *t*-test. Differences between the two types of continuous data that were not normally distributed were compared by the rank sum test, and differences between the two types of categorical data were compared by the chi-square test. All reported *P* values were two-sided, and a value of *P* < 0.05 was considered statistically significant. The cut-off points with diagnostic value are shown by the receiver operating characteristic (ROC) curve. A logistic regression analysis was used to analyze multiple factors that could produce the same outcome. In order to understand which factors contribute to the occurrence of adverse events, we use logistic regression and Cox regression analysis was used to analyze the events over time during the 3-years followed up.

## Results

### Baseline data

The patients’ baseline characteristics are shown in Table 1. There were no significant differences in sex, age, or height, but weight, body mass index (BMI), and body surface area (BSA) in the RH group were significantly higher than those in the control group (all *P*<0.05). In the RH group, the median office SBP, office DBP, GLS, GWI, GCW, GWW, and GWE were significantly higher than those in the control group (all *P* < 0.05).

**Table 1:** Comparison of baseline characteristics between the control group and the RH group

Variable	Normal (n = 283)	RH (n = 283)	*P* value
Female (%)	112 (39.2%)	96 (33.6%)	0.165
Age (y)	51.55 ± 13.40	51.61 ± 13.08	0.957
Heigh (m)	1.67 ± 0.86	1.67 ± 0.86	0.099
Weight (kg)	63 (55.95–70.25)	70 (60–78)	< 0.001
BMI (kg/m^2^)	22.97 (21.35–70.25)	24.91 (23.00–26.90)	< 0.001
BSA (m^2^)	1.65 (1.54–1.80)	1.78 (1.60–1.90)	< 0.001
Office SBP (mmHg)	122 (114–128)	150 (140–160)	< 0.001
Office DBP (mmHg)	78 (70–82)	95 (87.50–104.00)	< 0.001
GLS (%)	− 20( − 21 to − 19)	− 18 ( − 20 to − 16)	< 0.001
GWI (mmHg%)	2047.5 (1864.75–2241)	2272.5 (1996.75–2553.50)	< 0.001
GCW (mmHg%)	2224.5 (2043–2425.75)	2541 (2201.25–2810.00)	< 0.001
GWW (mmHg%)	94 (60–132)	134 (92.75–196.50)	< 0.001
GWE (%)	95 (93.75–97)	94 (92–96)	< 0.001

Data are presented as the mean ± standard deviation, number (%), or median (interquartile range)

*BMI* Body mass index; *BSA* Body surface area; *DBP* Diastolic blood pressure; *GCW* Global constructive work; *GLS* Global longitudinal strain; *GWE* Global work efficiency; *GWI* Global work index; *GWW* Global wasted work; *SBP* Systolic blood pressure

Statistically significant at *P* < 0.05

### Adverse events

According to the definition of adverse events mentioned above, adverse events included myocardial infarction, heart failure, renal insufficiency, renal failure, cerebral infarction, and cerebral hemorrhage. No death events occurred (Table 2).

**Table 2:** Type and proportion of adverse events

Adverse events	Number (n)	Ratio (%)
Renal insufficiency	40	48.78
Renal failure	2	0.02
Myocardial infarction	29	35.36
Heart failure	4	0.05
Cerebral infarction	5	0.06
Cerebral hemorrhage	2	0.02

Data are presented as the number (%)

### Comparison of data between the non-adverse events group and the adverse events group

Table 3 shows that the number of adverse events (n = 82) accounted for 28.98% of all enrolled patients with RH (n = 283), among which men accounted for 78%. More than half of the patients with adverse events had a family history of hypertension (82.9%) and LV wall remodeling (68.3%). The incidence of smoking was also higher (*P* = 0.008) in the adverse events group than in the non-adverse events group (43.9% vs. 31.9%), while only 47.1% of patients in the non-adverse events group had a family history of hypertension and 33.8% had LV wall remodeling. Laboratory indicators, such as blood glucose concentrations, were higher in the adverse events group than in the non-adverse events group (6.16 ± 1.84 vs. 5.71 ± 1.58 mmol/L, *P* < 0.05). The mean low-density lipoprotein concentration was lower in the adverse events group than in the non-adverse events group (1.59 ± 0.87 vs. 1.90 ± 1.61 mmol/L, *P* < 0.05), but high-density lipoprotein, total cholesterol, and triglyceride concentrations were not significantly different between the two groups. Furthermore, BMI, BSA, office SBP, office DBP, 24-h ambulatory BP (mean daily SBP, mean daily DBP, diurnal mean SBP, diurnal mean DBP, night mean SBP, and night mean DBP) and all MW-related indicators (GLS, GWI, GCW, and GWE) in the adverse events group were significantly lower than those in the non-adverse events group. However, GWW was significantly higher in the adverse events group than in the non-adverse event group (*P* < 0.05).

**Table 3:** Comparison of baseline characteristics between the no adverse events group and the adverse events group

Basic characteristics and results	No adverse events (n = 201)	Adverse events (n = 82)	*P* value
*Population composition and measurement*			
Female sex (%)	78 (38.8%)	18 (22.0%)	0.008
Age (Years)	51 (43.25–60)	49 (41.75–59.25)	0.236
Heigh (m)	1.67 (1.60–1.73)	1.69 (1.61–1.73)	0.185
Weight (kg)	69.25 (60–76)	75 (65–84)	0.06
BMI (kg/m^2^)	24.53 (22.90–26.31)	26.08 (23.41–28.35)	0.02
BSA (m^2^)	1.76 (1.58–1.89)	1.83 (1.65–1.95)	0.025
Office SBP (mmHg)	148 (140–160)	158 (145–170)	< 0.001
Office DBP (mmHg)	93.19 ± 12.95	99.9 ± 17.43	0.002
*Cardiovascular disease risk factors*			
Smoking history	65 (31.90%)	36 (43.90%)	0.008
Drinking history	78 (38.20%)	43 (52.40%)	0.054
Family history	96 (47.10%)	68 (82.90%)	0.028
LV^*^ remodeling	69 (33.80%)	56 (68.30%)	< 0.001
High blood glucose	72 (35.80%)	42 (51.20%)	0.021
*Laboratory tests*			
Glucose (mmol/L)	5.71 ± 1.58	6.16 ± 1.84	0.039
TC (mmol/L)	4.62 ± 1.14	4.72 ± 0.99	0.499
TG (mmol/L)	1.59 ± 0.87	1.90 ± 1.61	0.102
LDL (mmol/L)	1.59 ± 0.87	1.90 ± 1.61	0.037
HDL (mmol/L)	1.22 (1.05–1.38)	1.15 (0.93–1.38)	0.52
*24 h ambulatory blood pressure*			
Average daily SBP (mmHg)	142.32 ± 15.18	149.91 ± 16.92	< 0.001
Average daily DBP (mmHg)	87.64 ± 11.21	92.20 ± 12.67	0.003
Diurnal mean SBP (mmHg)	147.09 ± 16.61	153.98 ± 18.50	0.002
Diurnal mean DBP (mmHg)	90.69 ± 12.47	94.85 ± 12.80	0.012
Night mean SBP (mmHg)	134.25 ± 13.92	144.32 ± 16.94	< 0.001
Night mean DBP (mmHg)	158 (145–170)	90 (79.75–96)	< 0.001
*Myocardial work related index*			
GLS (%)	–18 (–20 to − 17)	− 16 (− 17.5 to − 14)	< 0.001
GWI (mmHg%)	2327 (2101–2611.5)	2079 (1824–2346)	< 0.001
GCW (mmHg%)	2610 (2295–2896)	2321 (2068–2600)	< 0.001
GWW (mmHg%)	127 (88–186.5)	161 (103–215.5)	0.022
GWE (%)	94 (92–96)	93 (91–94.5)	< 0.001

Data are presented as the mean ± standard deviation, number (%), or median (interquartile range)

*BMI* Body mass index; *BSA* Body surface area; *DBP* Diastolic blood pressure; *GCW* Global constructive work; *GLS* Global longitudinal strain; *GWE* Global work efficiency; *GWI* Global work index; *GWW* Global wasted work; *HDL* High-density lipoprotein; *LDL* Low-density lipoprotein; *LV* Left ventricular; *SBP* Systolic blood pressure; *TC* Total cholesterol; *TG* Triglycerides

Statistically significant at *P* < 0.05

**Fig. 1:**
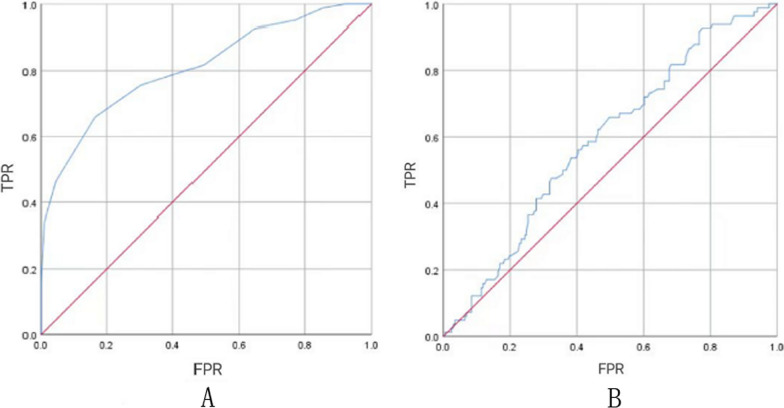
ROC curve about GLS (**A**) and GWW (**B**). FPR, false positive ratio; GLS, global longitudinal strain; GWW, global wasted work; ROC, receiver operating characteristic; TPR, true positive ratio

According to the ROC curve analysis of GLS (Fig. 1A) and GWW (Fig. 1B), − 16% was the cut-off value of GLS (sensitivity: 65.9%, specificity: 83.6%), and 127 mmHg% was the cut-off value of GWW (sensitivity: 65.9%, specificity: 50.2%).

The logistic analysis showed that the effect of LV remodeling on the incidence of adverse events was significant (odds ratio [OR] = 2.64, 95% confidence interval [CI] 1.26–5.51, *P* = 0.01). GLS with an absolute value of 16% as the cut-off value had a significant effect on adverse events (OR = 6.79, 95% CI 3.19–14.47, *P* < 0.001). GWW with 127 mmHg% as the cut-off value had a significant effect on adverse events (OR = 2.57, 95% CI 1.24–5.34, *P* = 0.01). Other factors, such as alcohol consumption (OR = 6.81, 95% CI 2.88–16.15, *P* < 0.001) and blood glucose concentrations (OR = 2.61, 95% CI 1.24–5.49, *P* = 0.01), also had a significant effect on adverse events (Table 4).

**Table 4:** Logistic analysis results

Variable	Group	B	B value standard error		Wald chi- square values		*P* value		OR	95% CI of OR	
Sex	Female										
	Male	− 0.16	0.53		0.09		0.77		0.85	0.30–2.43	
Age		− 0.02	0.02		1.03		0.31		0.985	0.96–1.02	
Smoking	No										
	Yes	0.14	0.44		0.10		0.76		1.15	0.48–2.74	
Drinking	No										
	Yes	1.92	0.44		18.99		< 0.001		6.81	2.88–16.15	
LV remodeling	No										
	Yes	0.97	0.38		6.68		0.010		2.64	1.26–5.51	
Glu (mmol/L)	Normal										
	High	0.96	0.38		6.33		0.01		2.61	1.24–5.49	
TC (mmol/L)	Normal										
	High	− 0.09	0.51		0.03		0.85		0.91	0.34–2.46	
TG (mmol/L)	Normal										
	High	− 0.09	0.39		0.06		0.81		0.91	0.42–1.97	
LDL (mmol/L)	Normal										
	High	0.25	0.54		0.22		0.64		1.29	0.45–3.73	
GLS (%)	Absolute value > 16										
	Absolute value ≤ 16	1.92	0.39		24.62		< 0.001		6.79	3.19–14.47	
GWW (mmHg%)	< 127										
	≥ 127	0.95	0.37		6.42		0.01		2.57	1.24–5.34	

*GLS* Global longitudinal strain; *Glu* Glucose; *GWW* Global wasted work; *LDL* Low-density lipoprotein; *LV* Left ventricular; *TC* Total cholesterol; *TG* Triglycerides control group

Statistically significant at *P* < 0.05

**Fig. 2:**
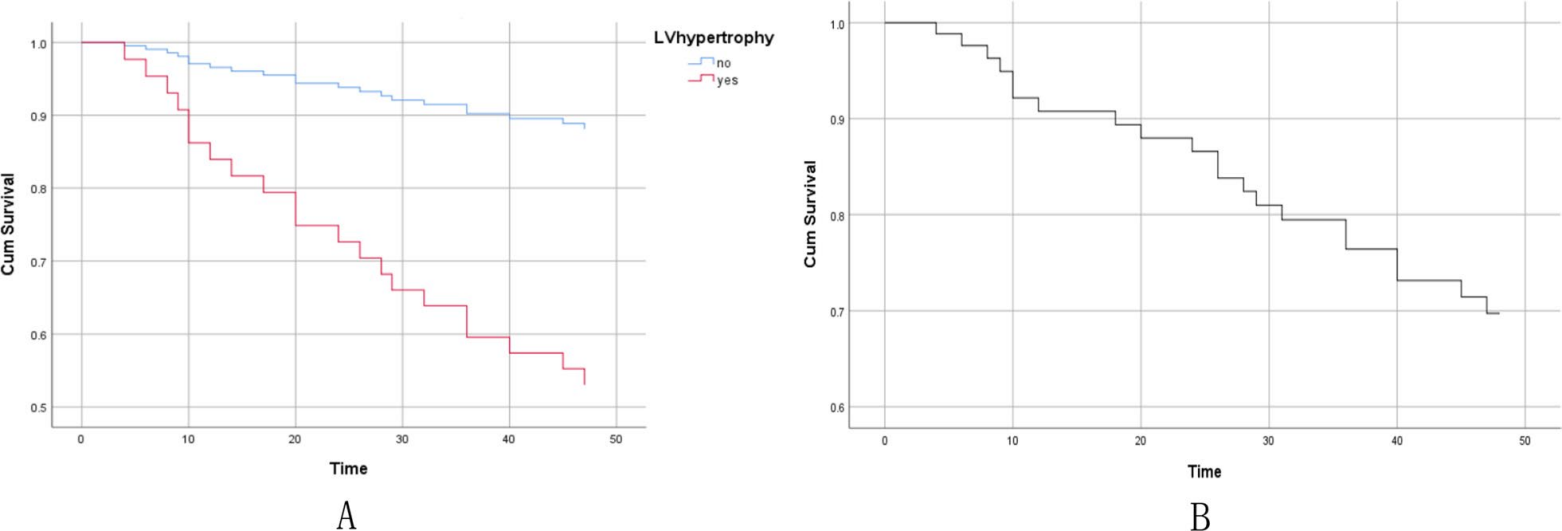
Cox analysis about the impact of LV hypertrophy (**A**) and SBP (**B**) to the adverse events occurring in 3 years. LV, left ventricular; SBP, systolic blood pressure

The Cox analysis showed that LV hypertrophy is an independent factor causing adverse events (hazard ratio, HR= 5.026, 95% CI 1.48–17.08, *P *< 0.05). Meanwhile, SBP also was a factor causing adverse events, SBP (HR=1.029, 95% CI 1.01–1.04, *P *< 0.001). (Fig 2.)

## Discussion

To the best of our knowledge, this is the first study to perform echocardiographic assessment of myocardial work to predict adverse events in patients with RH. In our study, all of the 283 enrolled patients underwent a complete echocardiographic examination and global MW analysis. We found the following results. (i) Echocardiography is still a safe and effective method to evaluate myocardial damage and cardiac contractile function, and can also be used to predict adverse events in patients with RH. (ii) Related indicators of MW were significantly different between patients with RH and adverse events and those without adverse events. Among these indicators, GLS, GWI, GCW, GWE in patients with adverse events were significantly reduced, and GWW was increased. (iii) Patients with a family history of hypertension developed high blood pressure (HBP) earlier, and LV remodeling occurred earlier and was more significant. If patients consumed alcohol, and had high blood glucose and high-density lipoprotein concentrations, adverse events were more likely to occur. The effect of hypertension on the heart is mainly achieved through the following three factors. (i) At the early stage of hypertension, the main damage is cardiac diastolic dysfunction, which increases LV filling pressure and heart cavity preload. High afterload caused by high BP then leads to LV eccentric or centripetal hypertrophy, and oxygen consumption to the subendocardial myocardium increases. Regardless of eccentric hypertrophy or centripetal hypertrophy, the myocardial alignment changes to some extent, and the interaction between cardiomyocytes also changes. This in turn affects effective myocardial contraction, and wasted work is increased. In addition, because of the heart resisting the high BP, myocardial contraction force is enhanced, and the lumen of the coronary arteries in the subendocardial myocardium is compressed and narrowed, eventually leading to myocardial ischemia. (ii) An increase in arterial BP leads to an increase in cardiac afterload to resist the increased BP and ensure cardiac output. Cardiomyocytes then increase in thickness, the coronary lumen collapses, and extravascular resistance increases, further leading to an increase in myocardial oxygen consumption. In addition, the thickened myocardium decreases the coronary blood flow reserve, leading to myocardial ischemia and hypoxia. (iii) Hypertension itself can lead to coronary artery stiffness, thereby causing myocardial ischemia and hypoxia. The result of the combination of myocardial ischemia and hypoxia causes myocardial fibrosis, myocardial uncoordinated contraction, and increased myocardial inactivity, which in turn acts on the heart, leading to cardiac hypertrophy and then heart failure.

Myocardial strain has been used to identify subclinical myocardial dysfunction in patients with hypertension for more than a decade. Among the different deformation (strain) components, longitudinal strain is important for predicting adverse events in patients with RH. Longitudinal strain corresponds to the function of the subendocardial layer of the myocardium in which longitudinal fibers are subjected to the negative effect of early development of fibrosis in hypertensive heart disease [23]. A histological analysis showed that the amount of subendocardial fibrosis was an independent determinant of longitudinal strain after adjusting for systolic wall stress [24]. Therefore, as subendocardial myocardial fibrosis increases, the effect on the longitudinal strain capacity of the myocardium increases. The longitudinal fibers located in the subendocardium are more susceptible to ischemia and are thus affected earlier in the ischemic cascade [25]. Myocardial contraction is closely associated with not only the ability of myocardial strain, but also with coronary flow and oxygen delivery. The balance between oxygen supply and demand is a critical determinant of the normal beat-to-beat function of the heart [26]. In patients with RH and long-term myocardial ischemia, the myocardium is gradually damaged, especially in the subendocardium, and the myocardial contractile stress is weakened. Based on these theories, when the myocardium is damaged, the strain generated by the subendocardial myocardium occurs first and most directly, which is manifested as a decrease in GLS. As we know, GLS is a semi-automated tool used to assess multidimensional myocardial mechanics, and it is more reproducible, and non-reliant on geometric assumptions [27], and is a strong predictor of outcome, particularly in individuals with a preserved ejection fraction [28]. In a study of 388 asymptomatic patients with hypertensive heart disease [29], the baseline GLS provided prognostic information that was independent and incremental over clinical parameters (age sex, heart rate, systolic BP, and atrial fibrillation) and concentric hypertrophy, and the optimal cutoff was − 16%. In our study, GLS also showed a good predictive performance for the occurrence of adverse events in 3 years in patients with RH, and − 16% was the cut-off value.

From the results of our study, GWW is another useful predictive index, it was higher in adverse events’ group. This finding may be related to long-term hypertension, leading to myocardial ischemia, especially the subendocardial myocardium, plays the main contractile role during the contraction process, leading to the wasted work increased. Slimani et al. assessed intraoperative myocardial histology and the stress–strain relationship in 101 patients with aortic stenosis (AS) who underwent aortic valve replacement [30], they found that, predictably, higher end-systolic stress led to lower LV GLS and circumferential strain, even after correcting for afterload, LV GLS, and LV GCW remained at or below the lower limit of normal. These results indicate that myocardial damage caused by myocardial ischemia is basically irreversible. From the level of MW, GLS-based myocardial work index, due to the irreversibility of GLS, leads to the decrease of myocardial GCW, while GWW has the opposite change. Our study also showed the increased GWW, RH patients with GWW > 127 mmHg% were more likely to have adverse events in 3 years.

Diabetes mellitus is an independent risk factor for coronary heart disease, and the proportion of patients with coronary heart disease can be as high as that in the diabetic population (55%) [31]. Coronary artery lesions in patients with diabetic coronary heart disease are complex and diffuse, and the degree of atherosclerosis is more serious than that without diabetes, which easily causes large-scale myocardial infarction, leading to hemodynamic instability and a poor prognosis. In our study, patients with RH and hyperglycemia were more likely to have adverse events. Previous reports have shown that alcohol consumption is associated with the development of hypertension [32–36]. In our study, alcohol consumption increased the risk of adverse events in patients with RH.

In conclusion, a family history of hypertension, hyperglycemia, hypertension itself, and LV hypertrophy eventually lead to abnormal coronary blood perfusion, and myocardial coronary artery perfusion is reduced. These series of events result in myocardial damage, decreased GLS, and increased GWW, which play an important role in adverse events in patients with RH.

### Limitations

This was a single-center study, and the enrolled subjects were limited to those who visited our hospital. Additionally, the number of RH patients included was not large and not sufficiently representative. Therefore, the results may only represent the population in the region where our hospital is located. There was also no group comparison by sex, and this remains to be further studied. The observation time was only 3 years, the data may be more meaningful if the follow-up period is extended. In addition, our criteria for the diagnosis of hypertension were SBP ≥140 mmHg and/or DBP ≥90 mmHg according to the American College of Cardiology/American Heart Association Guideline for the Prevention, Detection, Evaluation and Management of High Blood Pressure in Adults [37]. This guideline defines hypertension as SBP ≥130 mmHg and/or DBP ≥80 mmHg. We classified SBP between 130 and 140 mmHg, and DBP between 80 and 90 mmHg as normal. Therefore, whether our results are still appropriate according to the new standards of hypertension recommended by the American College of Cardiology/American Heart Association remains to be determined. As we know, MW is based on strain and BP, and strain is evaluated by the technique of 2D-STE. 2D-STE has its own limitation, for example, it depends on the temporal stability of tracking patterns and needs high quality grey-scale images for reducing inter- and intra-observer variability of tracking data. Another major limitation of 2D-STE methodology is the lack of standardisation, due to a relevant intervendor variability [38]. In addition, recent evidences have highlighted the possible influence of the chest wall conformation on the cardiac kinetics and deformation indices [39].

## Conclusions

Adverse events in patients with RH are the result of a combination of multiple factors. Patients with a family history of hypertension, combined with hyperglycemia, LV wall remodeling, and alcohol consumption, are more likely to have adverse events within 3 years. GLS and GWW are not only factors affecting the occurrence of adverse events but also reliable indicators for predicting the occurrence of adverse events, such as the absolute value of GLS < 16% and GWW > 127 mmHg%.

## Data Availability

The authors confirm that the data supporting the findings of this study are available within the article.

## Abbreviations

AS: Aortic stenosis
BMI: Body mass index
BP: Blood pressure
BSA: Body surface area
DBP: Diastolic blood pressure
FPR: False positive ratio
GCW: Global constructive work
GLS: Global longitudinal strain
GWI: Global work index
GWW: Global wasted work
GWE: Global work efficiency
HBP: High blood pressure
IQR: Interquartile range
IVR: Isovolumic relaxation
LV: Left ventricular
MW: Myocardial work
OSA: Obstructive sleep apnea
RH: Resistant hypertension
ROC: Receiver operating characteristic
SBP: Systolic blood pressure
STE: Strain by speckle-tracking echocardiography
TPR: True positive ratio
TTE: Transthoracic echocardiography
